# Supplementary material for: Suppression of the alpha, delta, and omicron variants of SARS-Cov-2 in Taiwan
Source: PLoS One. 2024 Mar 18;19(3):e0300303. doi: 10.1371/journal.pone.0300303 (PMC10947699; doi:10.1371/journal.pone.0300303)
Supplement: S1 Appendix — (DOCX) [file pone.0300303.s001.docx]

**Appendix**

**Suppression of the Alpha, Delta, and Omicron variants of SARS-Cov-2 in Taiwan**

Supporting Information **1. Border control measures**

The Oxford COVID-19 Government Response Tracker (OxCGRT) [1] gathers publicly accessible data on 24 indicators spanning government actions in the following four dimensions: (1) containment and closure, (2) economic response, (3) health systems, and (4) vaccine policies. Within the category of "containment and closure," specific indicators include the school closing (C1), workplace closing (C2), cancel public events (C3), restrictions on gatherings (C4), close public transport (C5), stay at home requirements (C6), restrictions on internal movement (C7), and international travel controls (C8). The international travel controls (C8) indicator is particularly used to assess border control measures. The coding for C8 is as follows: 0 - no restrictions, 1 - screening arrivals, 2 - quarantine arrivals from some or all regions, 3 - ban arrivals from some regions, 4 - ban on all regions or total border closure, and Blank - no data. This indicator provides valuable information on the extent of government measures in controlling international travel, contributing to a comprehensive understanding of a country's response to the COVID-19 pandemic.

We compare the epidemic outcomes-related performance of Taiwan and other island countries. Figure S1 depicts the implementation of border control measures by island countries in response to the emergence of the first COVID-19 case in China, highlighting Taiwan's rapid deployment of border control measures. Following the implementation of border control measures, Taiwan recorded its first confirmed COVID-19 case after a 21-day interval. Despite the increase in the cumulative number of deaths per million people since May 2022, Taiwan's overall performance remains commendable when compared to other island countries, based on data from Our World in Data [2]. (Figure S2)

***Supplementary Figure S1.* The time to any border closure from first reported case in China and reference country.**


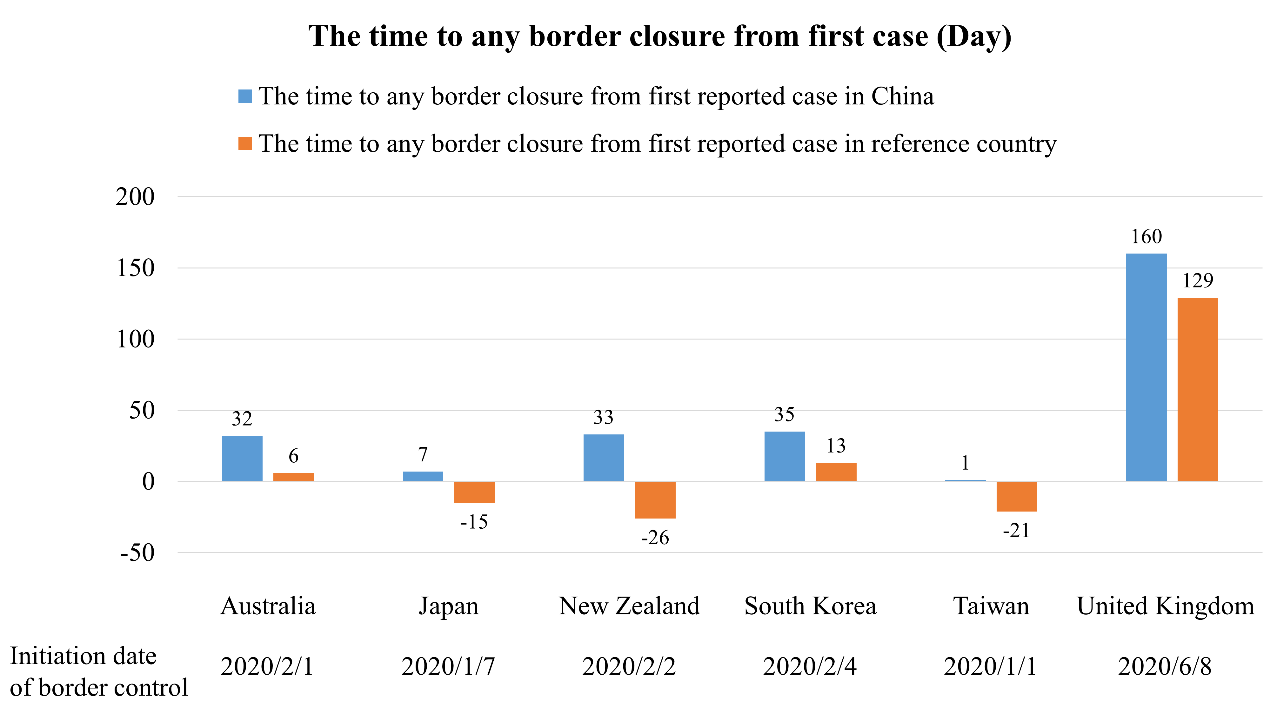


***Supplementary Figure S2.* Cumulative deaths per million people in 2020-2022.**


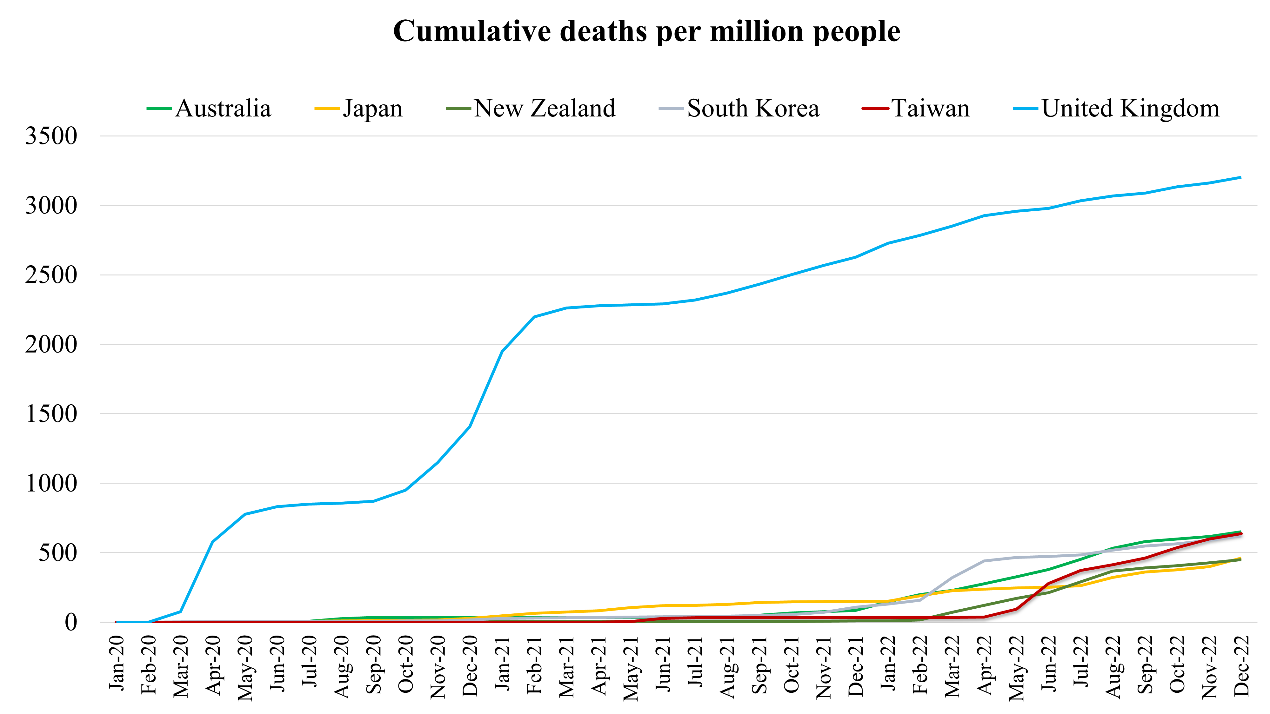


Supporting Information **2. Time-varying reproduction number**

The time-varying reproduction number $R_{t}$ can be estimated using method of Cori et al. It is estimated by the ratio of number of new infections generated at time t. $I_{t}$ is the total infectiousness of infected individuals at time t. $w_{s}$ is a generation in interval or probability that *s* day separate the moment of infection in an index case and a daughter case. The formula of $R_{t}$ by method of Cori et al. is

$$R_{t}=\frac{I_{t}}{\sum_{s=1}^{t} w_{s}I_{t-s}}.$$

$R_{t}$ is also consider the average number of secondary cases that each infected individual would infect if the conditions remained as they were at time t. The corresponding package in R for evaluating Rt is called EpiEstim (https://cran.r-project.org/web/packages/EpiEstim/index.html). In this package, we used the function, called “estimate_R”, with the method of "parametric_si" to evaluate Rt. The information of serial interval is mean = 2.9 and sd = 1.5. The information of confirmed case is in file “221020 Number of confirmed cases.csv”.

Supporting Information **3. Quarantine measures and compensation**

On May 15, 2021, the CECC raised the epidemic warning level for Taipei City and New Taipei City to a Level 3 alert [3], followed by a Level 3 alert nationwide on May 19 [4]. People in Taiwan were requested to wear masks in public transportation as early as April 2020 [5]. After the May 2021 Level 3 alert, the CECC imposed immediate control measures requiring people to wear masks in indoor and outdoor public spaces. In response to the rapid spread of the delta variant worldwide, the CECC implemented longstanding measures, such as quarantine and isolation, to contain COVID-19 transmission [6]. They began offering anyone who had tested positive the option of quarantine in a government-provided facility. The provision of isolation facilities reduced the spread of the virus within families greatly, thereby reducing the number of cases in the community.

People who break quarantine regulations are fined, whereas those who comply are compensated. All citizens or holders of resident certificates who complied with regulations can apply for $35 per day quarantine compensation [7]. Moreover, the Tourism Bureau in Taiwan has implemented a subsidy plan for hotels that collaborate with the government to provide rooms for people subject to quarantine. Each hotel offering rooms for quarantine could receive a subsidy of $35 per room per day from April 1 to July 31[8]. To comply with the government’s quarantine policy, 13% of hotels (25,609 of 197,618 hotels) in Taiwan were converted into hotels for quarantine, likely facilitating Taiwan's success in epidemic prevention [9,10].

Supporting Information 4. Google mobility reports^11^

The figures show movement trends within Taiwan, across different categories of places. The data shows how visitors' presence in categorized places changed compared to baseline (pre-alert) days. The baseline day is the median value from the 5‑week period of January 3–February 6, 2020, representing a normal value for that day of the week. The x-axis shows the dates and the y-axis represents the percent change from baseline.

***Supplementary Figure S3.* Community mobility in Taiwan from January 1 to February 20, 2022.**


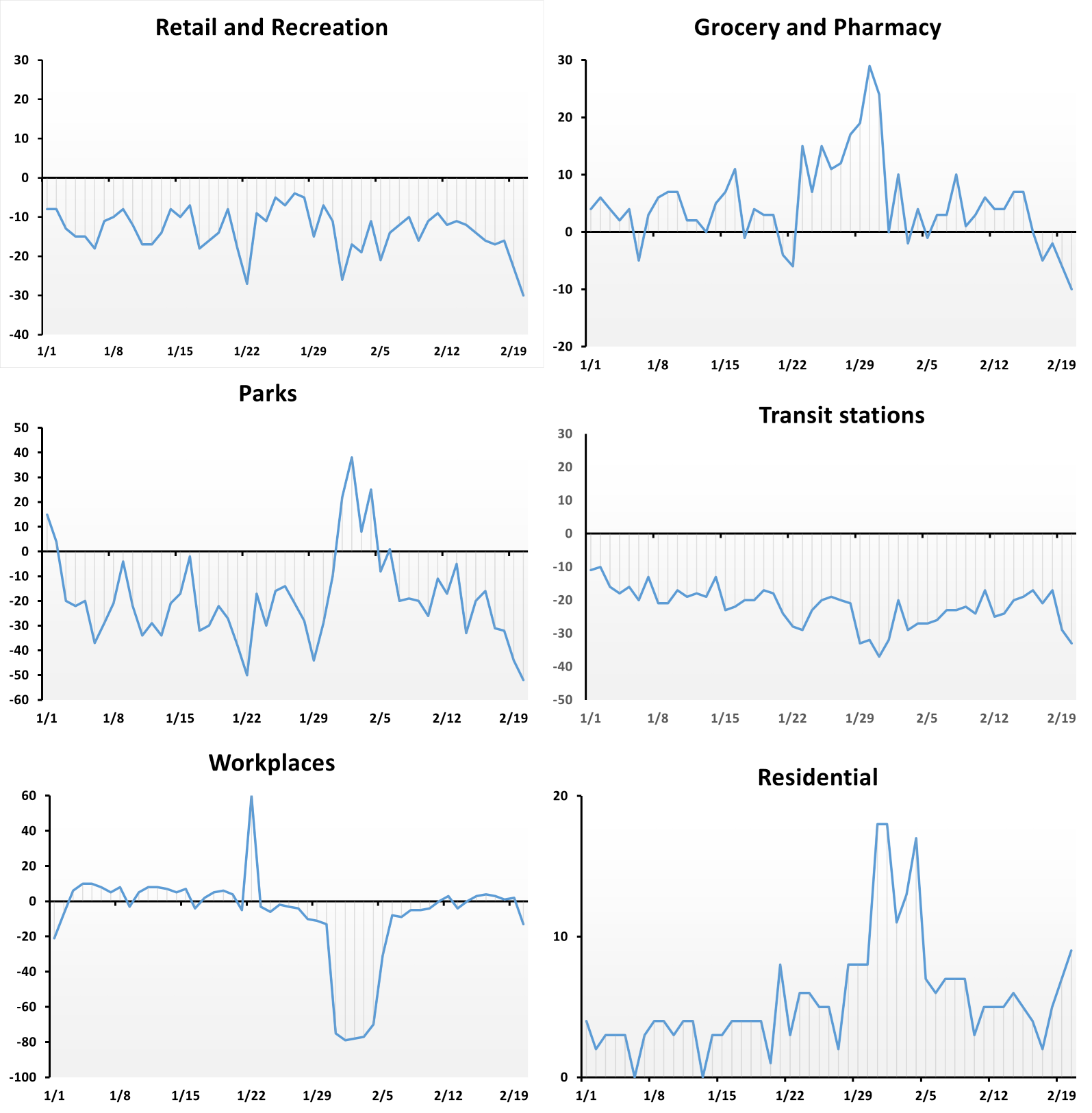


***Supplementary Figure S4.* The change in human mobility* in Taiwan before and after the Level-3 COVID-19 alert issued on May 15, 2021.** (a) The average number of daily trips from May 3–9. (b) The average number of daily trips from May 17–23.

*Human mobility: daily mobile phone data obtained from Far EasTone Telecommunications in Taiwan.

**
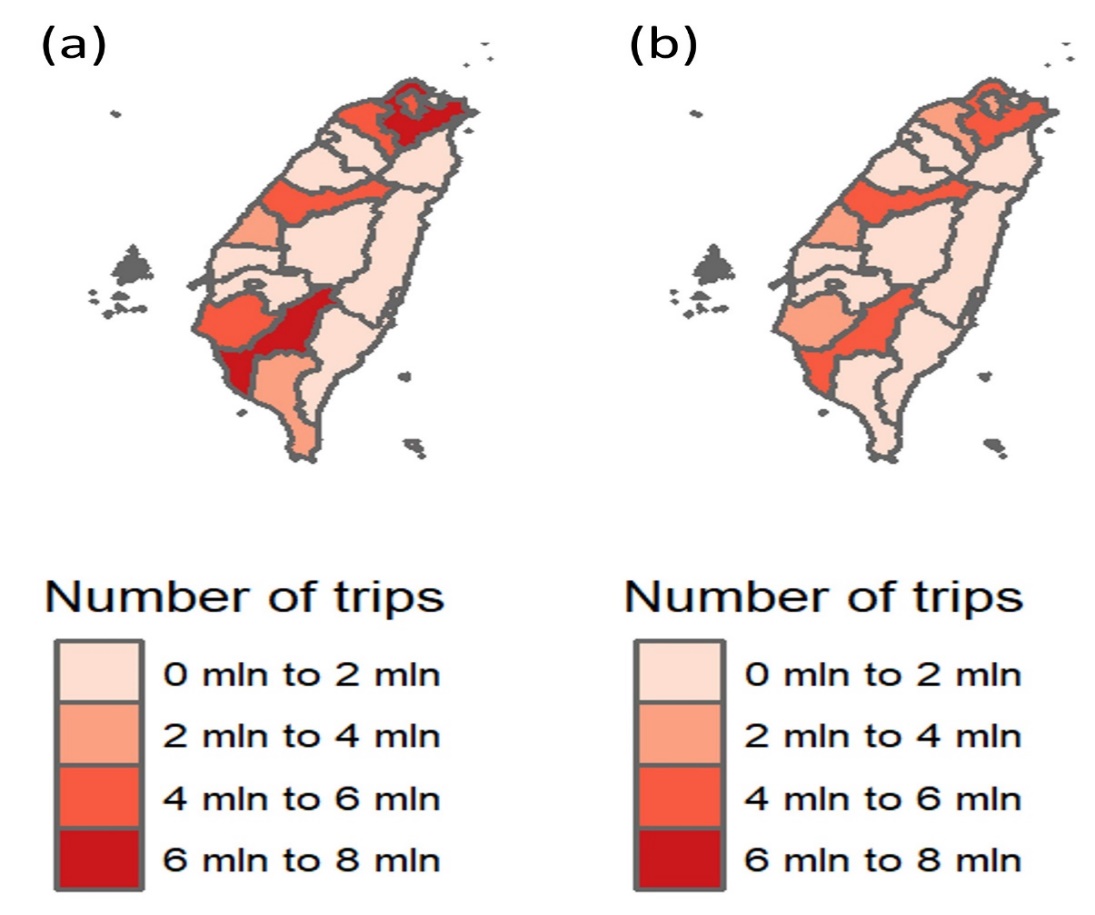
**

Supporting Information 5. Unity and cooperation

The data was obtained via the Imperial College London YouGov Covid-19 Behaviour Tracker [12], which is a global anonymized respondent-level biweekly survey. We extracted the 24~28 locations that were surveyed in September 2020. Participants were questioned about three categories: (i) sense of social unity, (ii) citizen satisfaction, and (iii) confidence in government. For question (i), except for Hong Kong (N=497), Japan(N=498), South Korea(N=458), and the Netherlands(N=502), the respondents' number in other countries was approximately 1000. For the last two questions, there was no data available for Hong Kong, Saudi Arabia, Thailand, and United Arab Emirates. For question (ii), except for Japan (N=850), South Korea(N=852), Netherlands(N=922), and the United Kingdom (N=3889), the respondents' number in other countries was approximately 2000. For question (iii), except for Japan (N=971), South Korea(N=852), Netherlands(N=974), and the United Kingdom (N=4023), the respondents' number in other countries was approximately 2000. To assess the sense of social unity, our research used the self-reported question: “Is your country now more united or more divided than before the coronavirus (COVID-19)? outbreak?”. Responses included “More united”, “More divided”, and “No change”. To estimate citizen satisfaction, we used “How well or badly do you think the Government are handling the issue of the Coronavirus (COVID-19)?”. The possible responses are “1 = Very well”, “2 = Somewhat well”, “3 = Somewhat badly”, “4 = very badly” and “5 = Don’t know”. To estimate confidence in government, we used “How much confidence do you have in the NHS to respond to a Coronavirus (COVID-19) outbreak in the UK?” (as an example of the UK). The responses are “1 = A lot of confidence”, “2 = A fair amount of confidence”, “3 = Not very much confidence”, “4 = No confidence at all” and “5 = Don’t know”. The results were shown in Figures S5-S10.

***Supplementary Figure S5.* Sense of Social Unity in 28 countries**

**
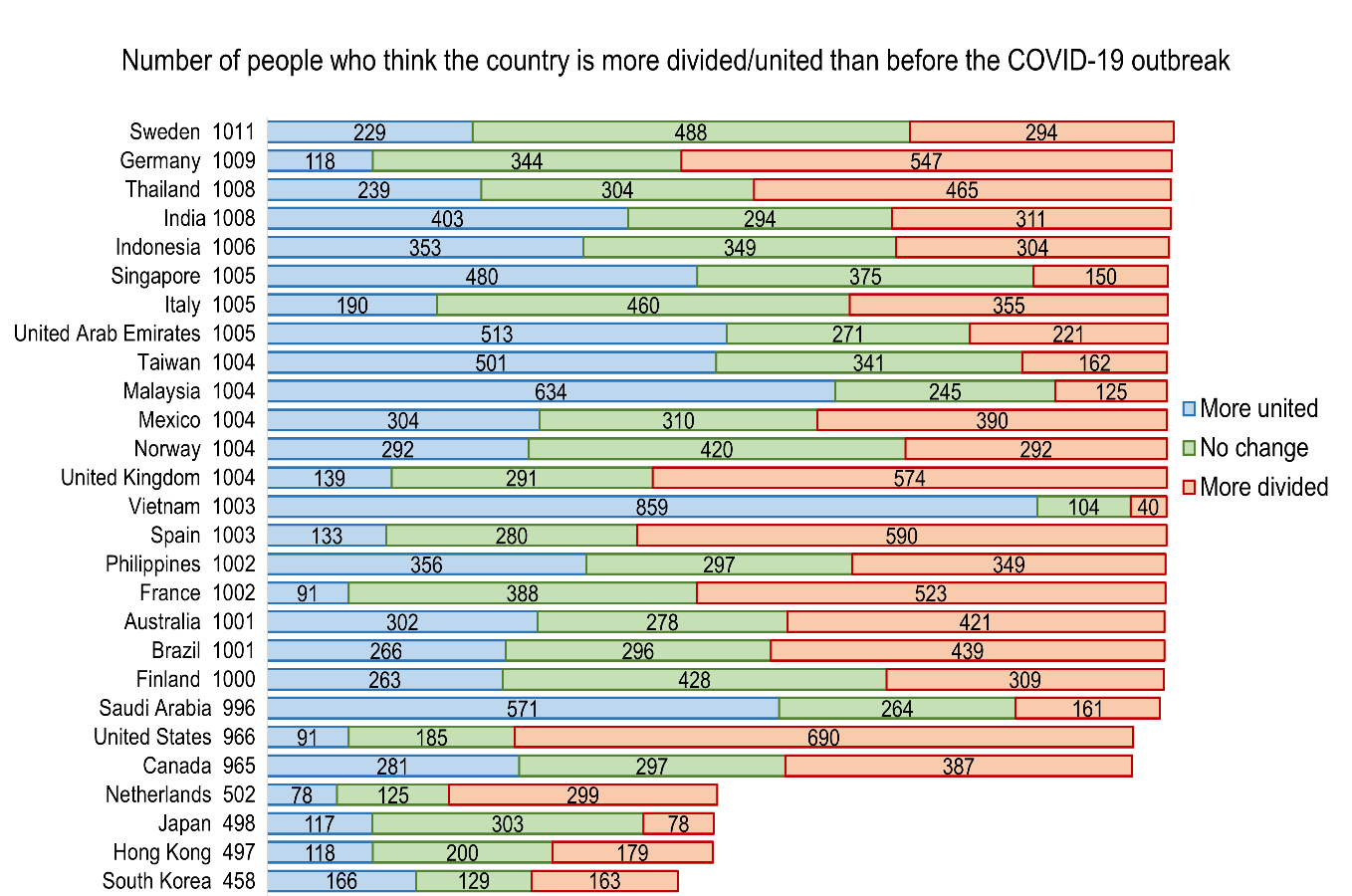
**

***Supplementary Figure S6.* Sense of Social Unity in 28 countries, Asia-Pacific and Overall**


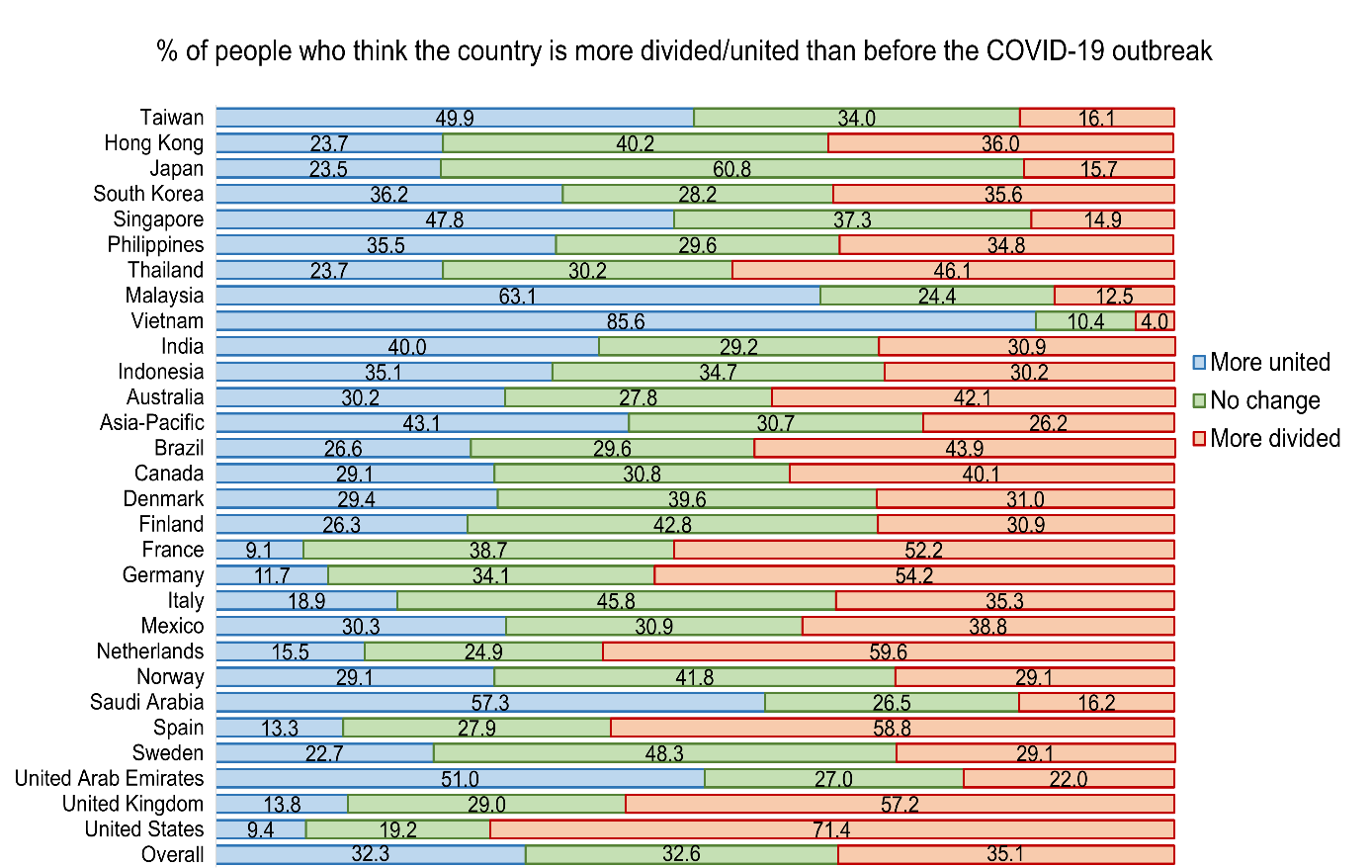


***Supplementary Figure S7.*** **Citizen satisfaction in 24 countries**


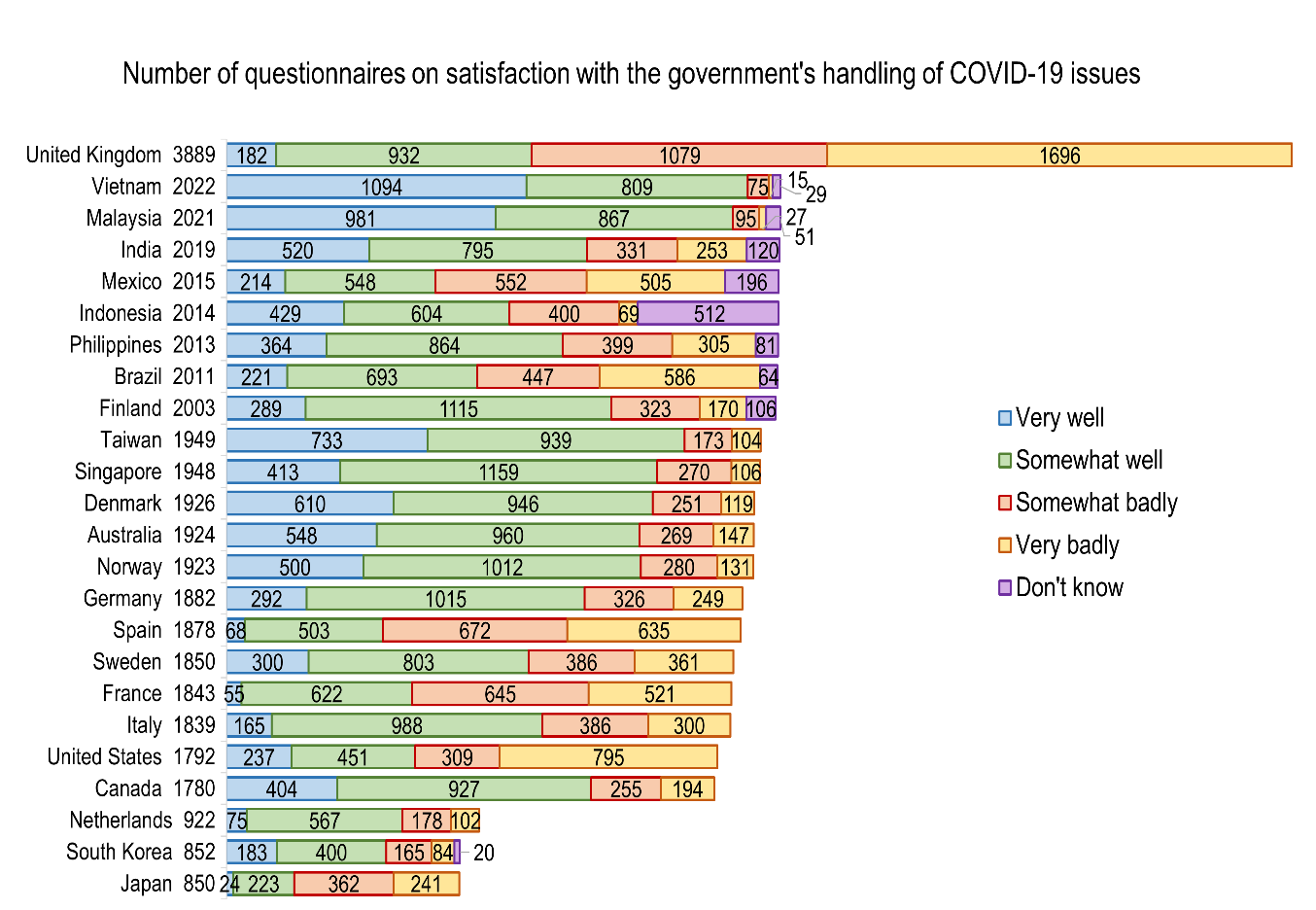


***Supplementary Figure S8. Citizen Satisfaction in 24 countries, Asia-Pacific and Overall***


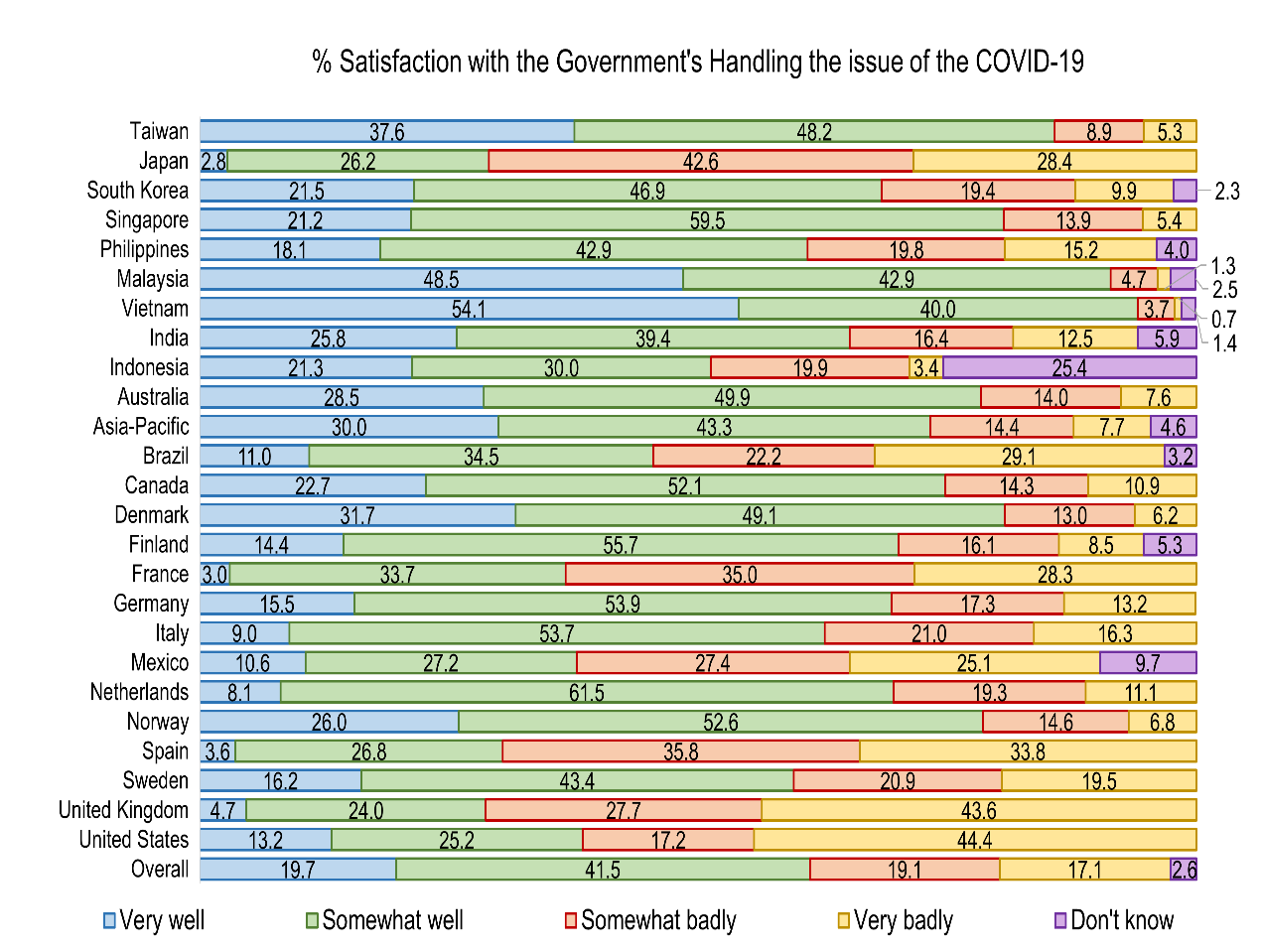


***Supplementary Figure S9.* Citizen Confidence in 24 countries**


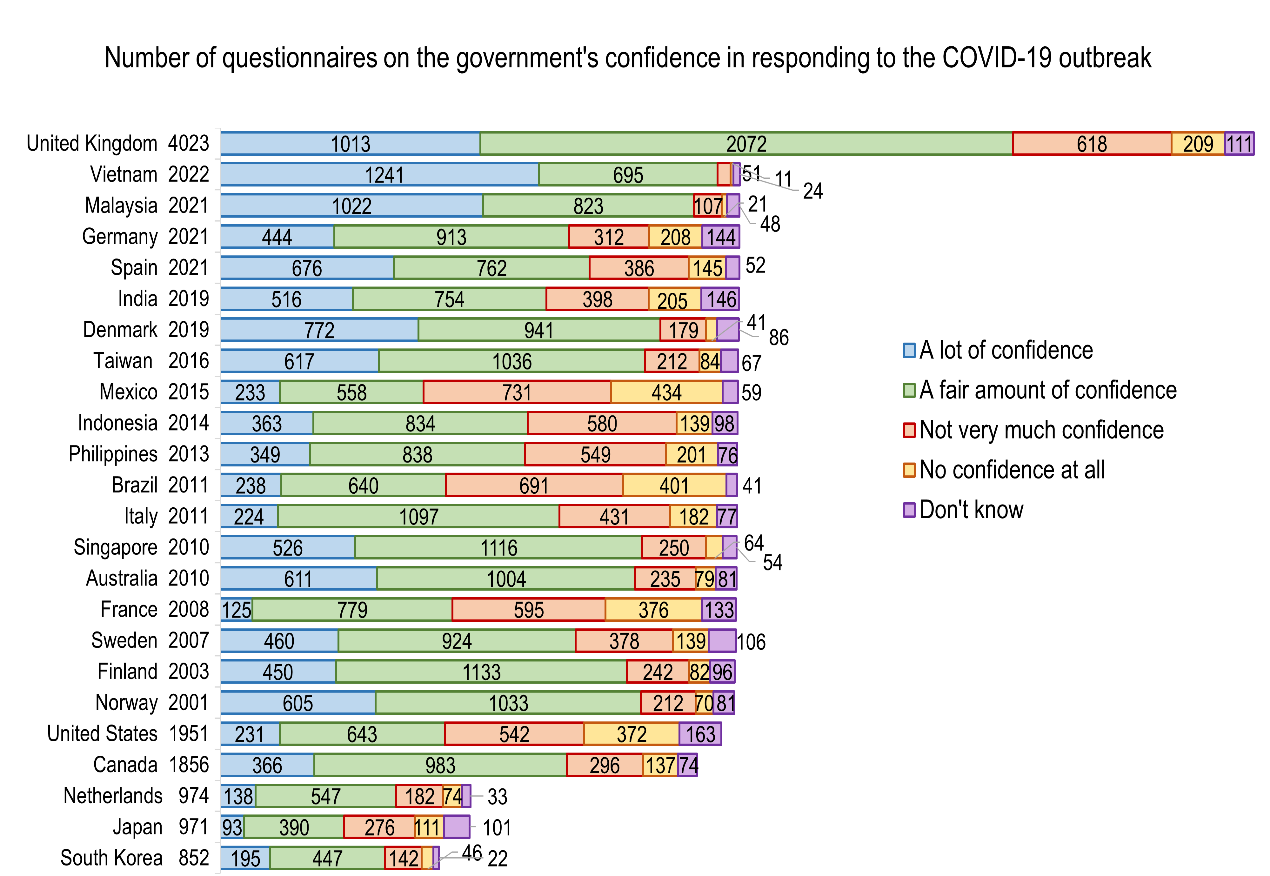


***Supplementary Figure S10.* Citizen Confidence in 24 countries, Asia-Pacific and Overall**

**
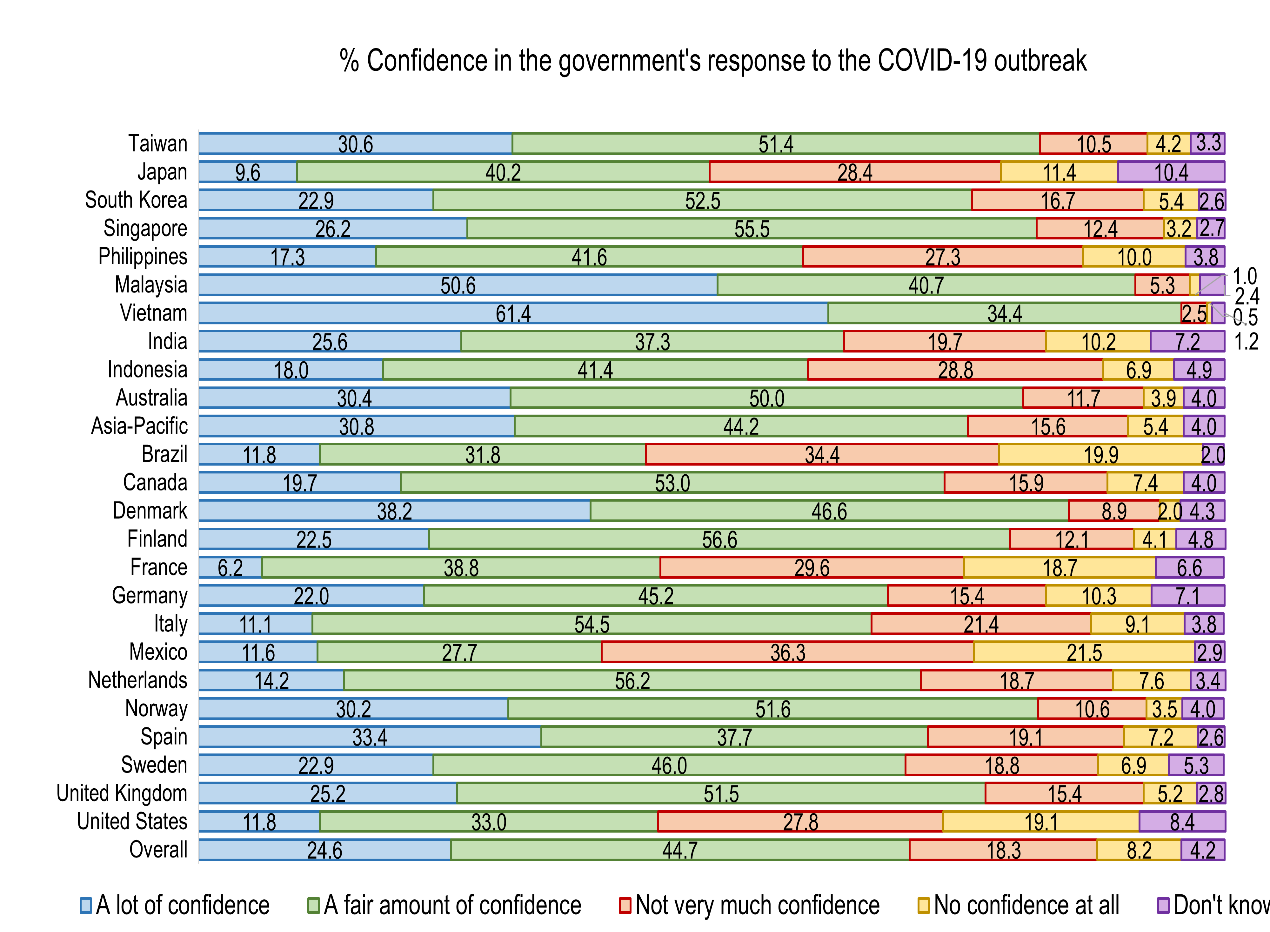
**

Supporting Information 6. Restrictions and control measures of epidemic alert level 3

The Central Epidemic Command Center (CECC) in Taiwan raises epidemic alert level for Taipei City and New Taipei City to Level 3 and strengthens national restrictions and measures, effective from May 15 to May 28, in response to increasing level of community transmission [13]. Main measures imposed under a Level 3 epidemic alert were listed below

1. Wear a mask outdoors

2. Closure of leisure and entertainment venues

3. Religious rites and rituals: a halt to all pilgrimages and processions associated with temples or churches. Other events should ensure contact-information registration, social distancing and heightened disinfection measures

4. Campuses in elementary and middle schools to be closed to the public

5. Avoid unnecessary movement, activities or gatherings, all family or social gatherings involving over 5 people indoors or 10 people outdoors are suspended

6. self-health monitoring

7. Business venues and places where personal business is conducted should implement crowd and flow control, enforce the wearing of masks and social distancing

8. Food and beverage vendors should use epidemic prevention measures, such as contact-information registration, social distancing and dividers. Those that can not adopt those measures are urged to offer take-out services.

9. Working in multiple offices, distance working and flexible working hours

10. enhanced disinfection of public places and public transport

Measures concerning the medical response systems in the areas under a Level 3 epidemic alert include the following:

1. Expansion of dedicated wards

2.Traffic control bundling and patient ward segregation

3. Outdoor testing posts

4. Widespread use of distance consultations and medical treatment

5. Proactive setup of designated and responding hospitals

People in the Level 2 and 3 areas are urged to reduce unnecessary movement between areas.

Supporting Information 7. Vaccination coverage

In 2021, more than 100 countries around the world began to administer COVID-19 vaccines. According to the national data published on the "Our World in Data" website [14], as of June 7, 2021, vaccination coverage (doses administered per person) was 100.87% in the UK, 90.97% in the USA, 74.48% in Singapore, 21.05% in South Korea, 18.17% in Japan, 6.63% in Thailand, 3.04% in Taiwan, and 1.37% in Vietnam, respectively.

Prior to May, 2021 these above discussed outbreaks, most Taiwanese people did not feel a great urgency for vaccination owing to the belief that there was no community spread of the virus on the island. As shown in Figure 1, the vaccination rate was extremely low before May. When the biggest COVID-19 outbreak occurred in mid-May, people became worried about the shortage of vaccines and thus became eager to get vaccinated. Facing a global vaccine shortage, the Taiwanese government has cooperated with companies and non-governmental organizations to obtain vaccines and thus alleviate its citizens' sense of urgency and anxiety about lack of vaccination. Vaccination coverage in Taiwan has increased steadily from 0.78% on May 14 to 45.94% on August 31, 2021.

Supporting Information 8. Delta variant

SARS-CoV-2 variants of concern (VOC) are most likely to evolve in regions with high virus transmission and low vaccine coverage [15]. According to the World Health Organization [16], a VOC is a variant that is associated with increased transmissibility, increased virulence, reduced neutralization by antibodies (induced by prior infection or vaccination), reduced diagnostic detection (i.e., more false negatives), or a disease course that is less hampered by treatments and/or vaccines. The alpha (B.1.1.7) variant was the first VOC to exhibit higher transmissibility than the ancestral strain, and it became the dominant VOC quickly in January 2021 [17]. The delta variant's mutations have made it bind human cells more efficiently, thus increasing it transmissibility and leading it to become by far the most dominant SARS-CoV-2 variant worldwide as of the writing of this paper [15].

Supporting Information 9. The Economic Impact of Taiwan's COVID-Zero Strategy

***Supplementary Figure S11.*** **The distribution of cumulative deaths per million people against unemployment rate in December 2020.**

**
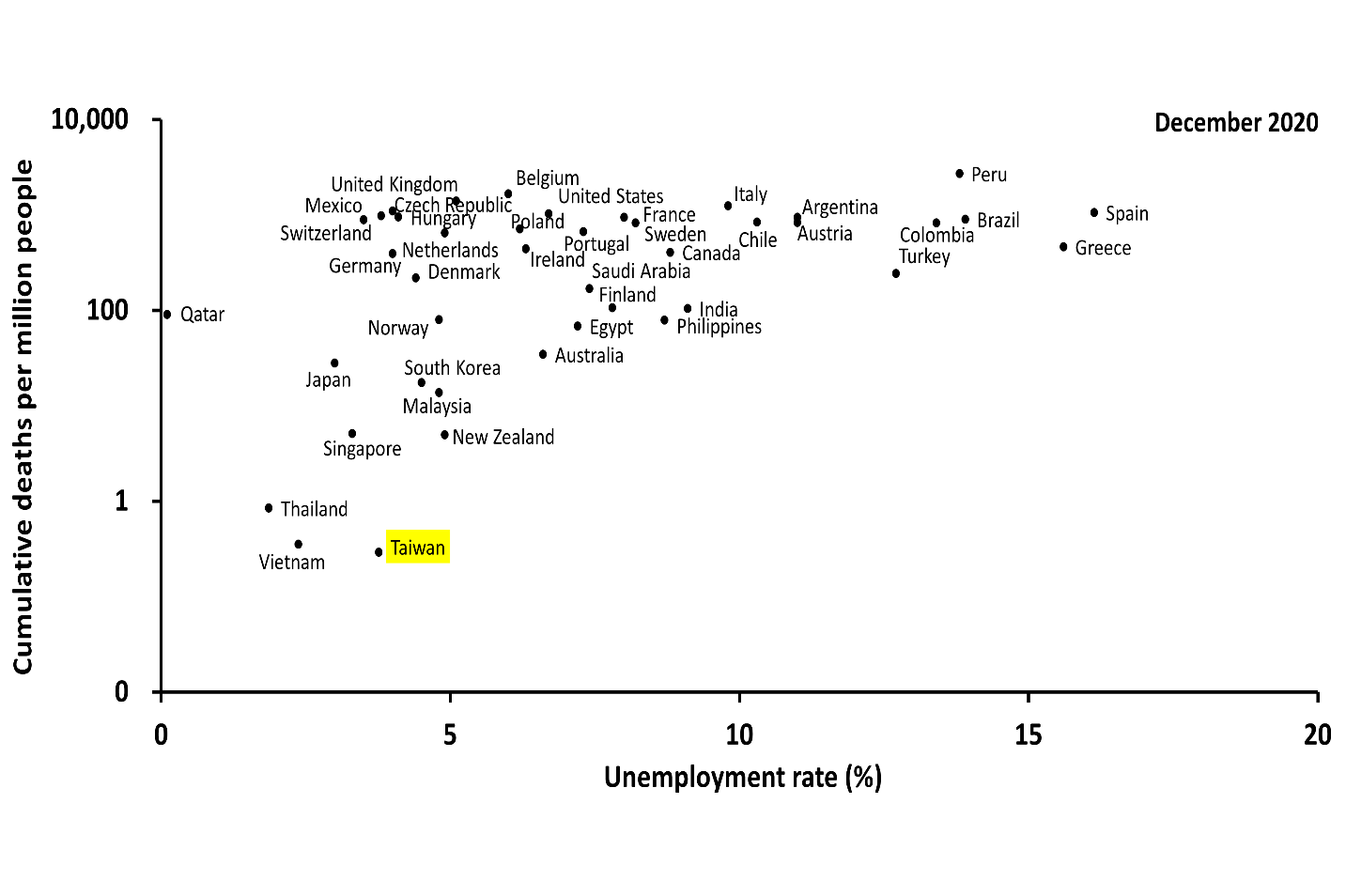
**

***Supplementary Figure S12.*** **The distribution of cumulative deaths per million people against unemployment rate in November 2021.**


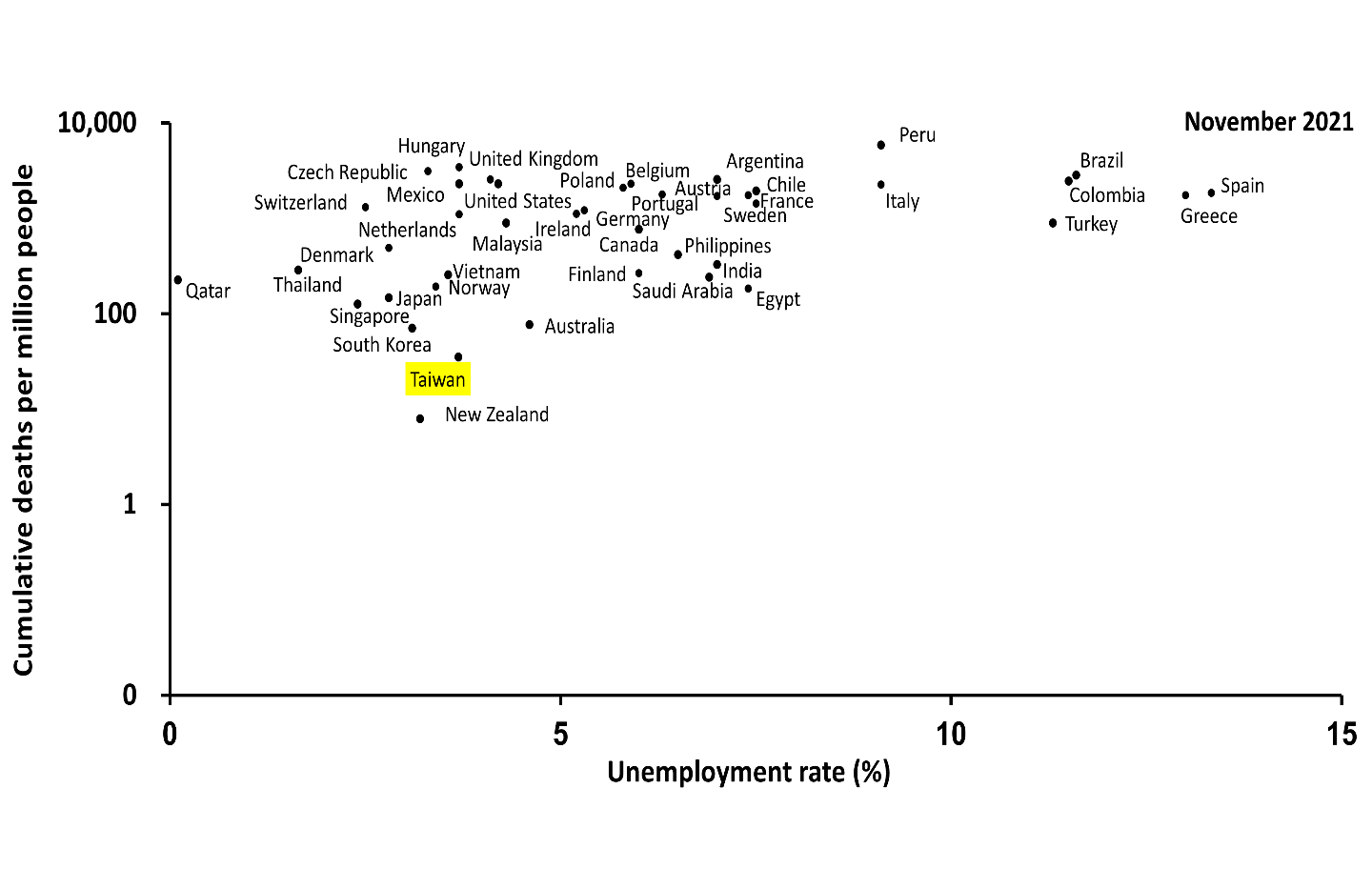


***Supplementary Figure S13.*** **The distribution of cumulative deaths per million people against GDP in December 2020.**


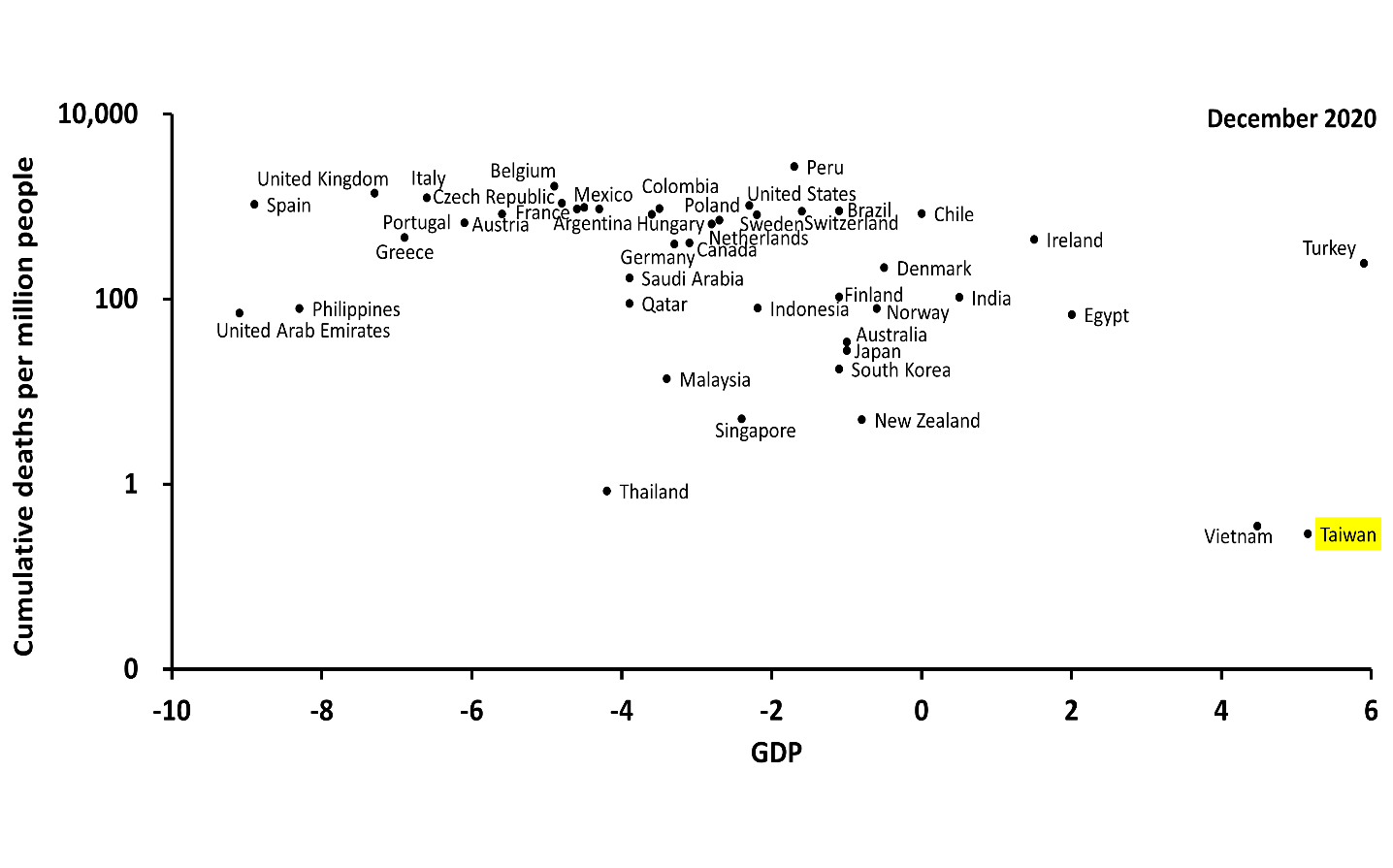


***Supplementary Figure S14.*** **The distribution of cumulative deaths per million people against GDP in November 2021.**


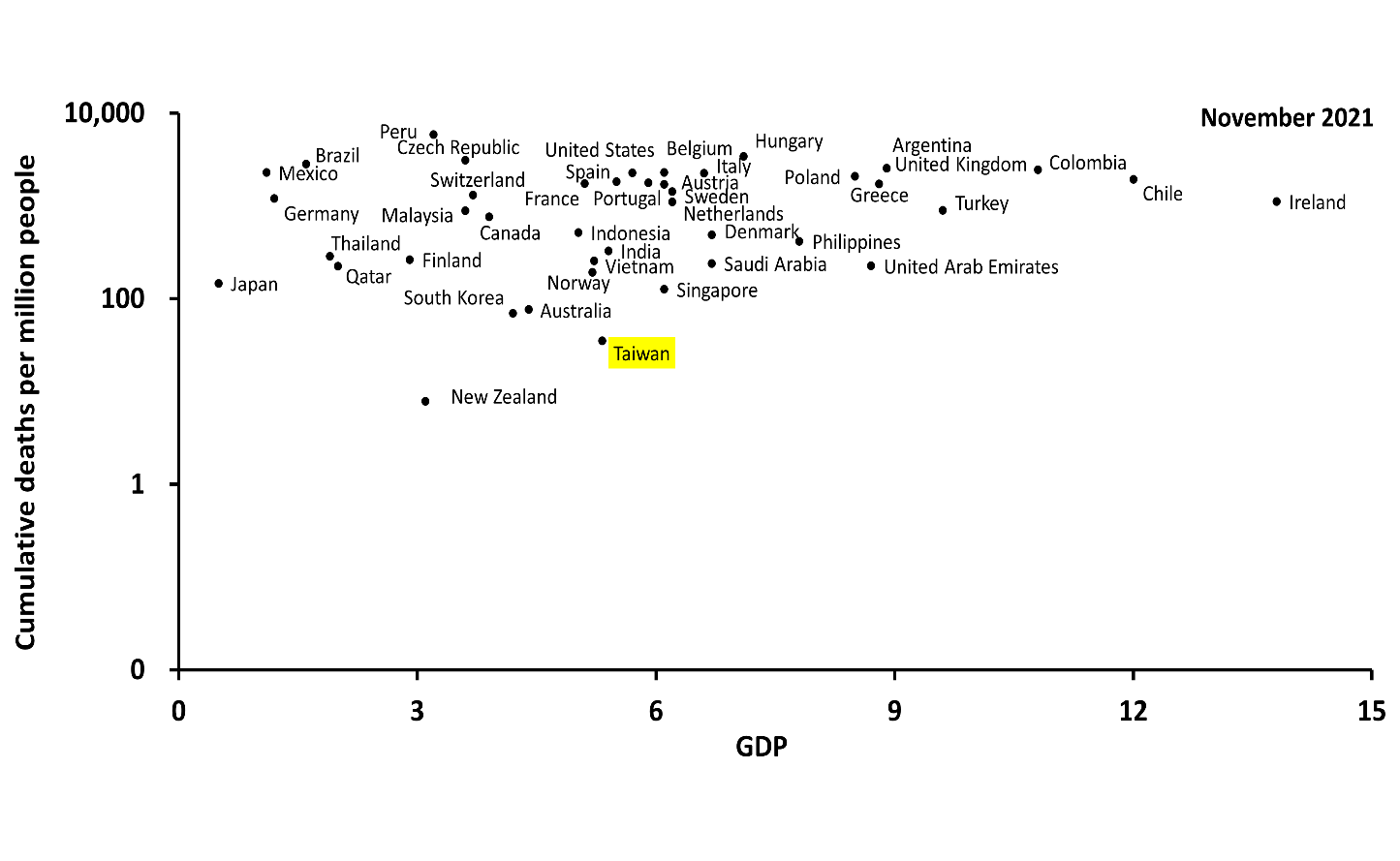


Supporting Information 10. Vaccination support and vaccine donation

Vaccination is a key method to prevent the spread of SARS-CoV-2 (including VOCs). In the absence of adequate vaccines globally, the Taiwanese government has cooperated with the private sector to make every effort to obtain vaccines. Many countries donated vaccines to Taiwan. Taiwan is very grateful for receiving vaccination support from many countries around the world (Figure S11) [18]. Besides, Taiwanese enterprises and private organizations joined the vaccine donation. The Taiwanese government is also very grateful for the help and donation from enterprises and private organizations. (Figure S12) [18].

***Supplementary Figure S15.* THANK YOU card from Taiwan.** Taiwan is very grateful for vaccine donations from many countries in the world.


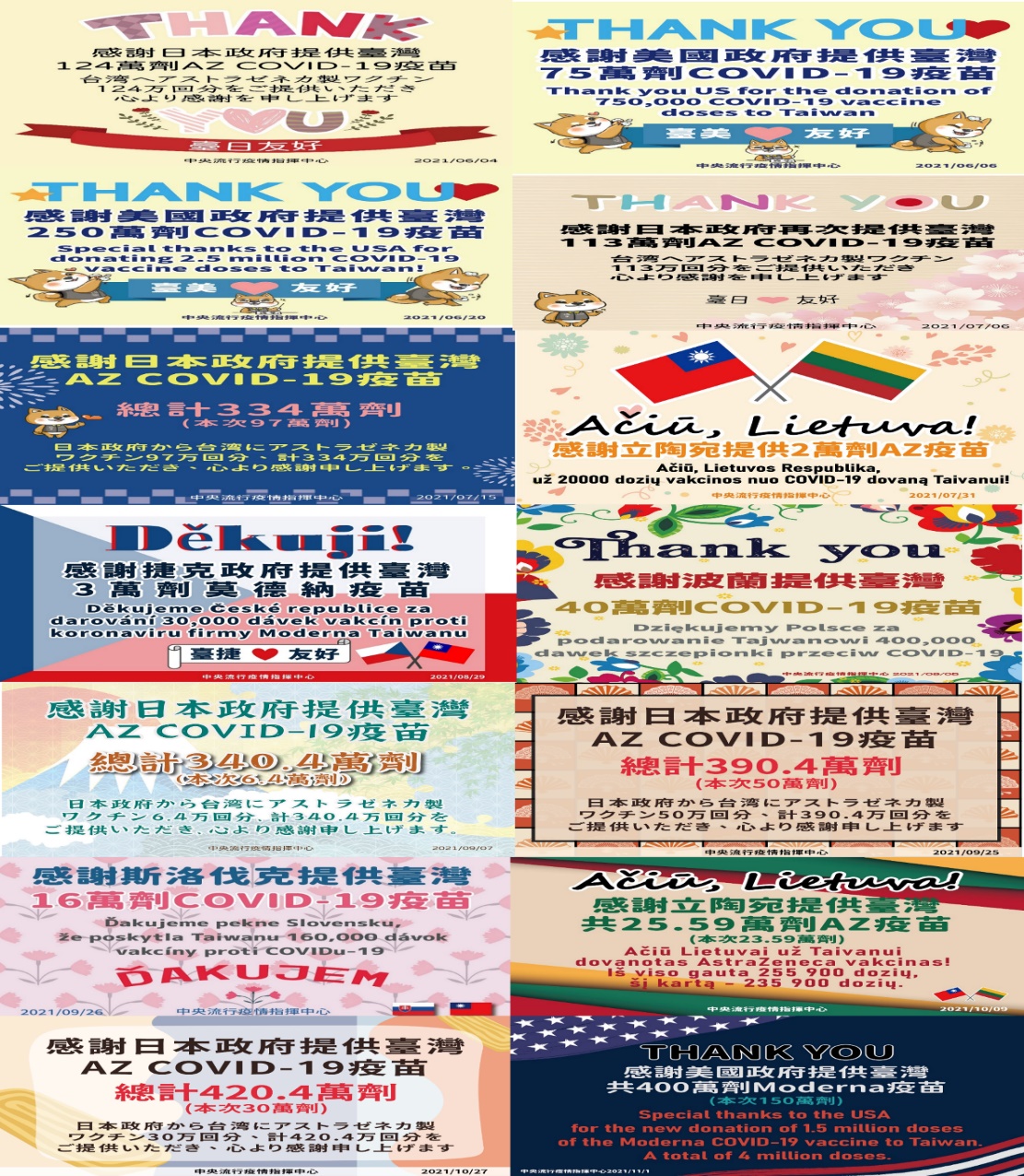


***Supplementary Figure S16.*** **THANK YOU card for Taiwanese enterprises and private organizations.** The Taiwan government is very grateful for vaccine donations from enterprises and private organizations.


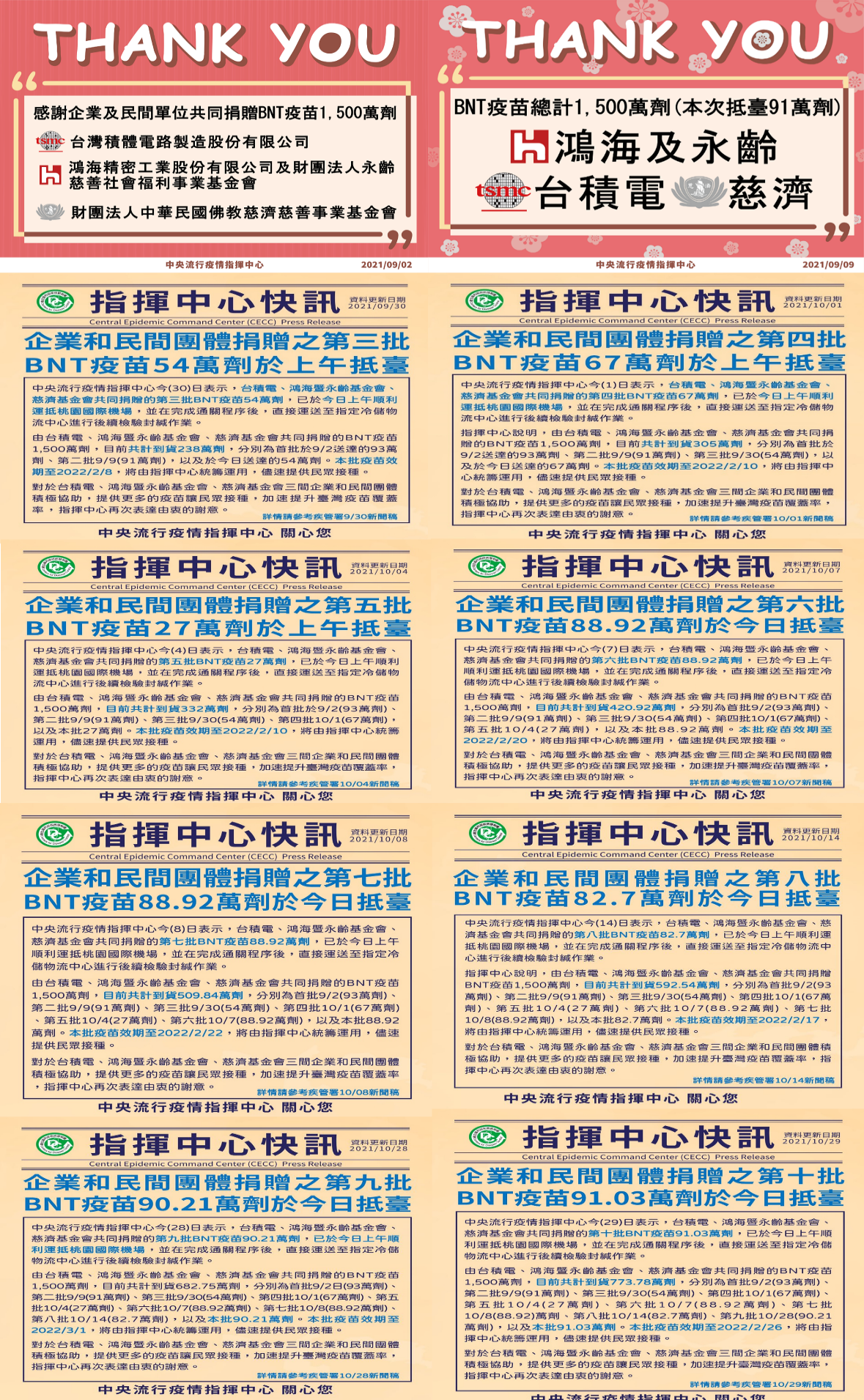


**Supplemental References**

1. OxCGRT. Coronavirus government response tracker [Internet]. University of Oxford. 2021 [Cited 2021 September13]. Available from: <https://www.bsg.ox.ac.uk/research/research-projects/coronavirus-government-response-tracker>.
2. Our World in Data. Coronavirus Pandemic (COVID-19) [Internet]. 2022 [Cited 2022 August 11]. Available from: https://ourworldindata.org/coronavirus-source-data
3. Taiwan Center for Disease Control (CDC). CECC raises epidemic alert level for Taipei City and New Taipei City to Level 3 and strengthens national restrictions and measures, effective from May 15 to May 28, in response to increasing level of community transmission [Internet]. Taiwan CDC. 2021 [Cited 2021 September 07]. Available online: https://www.cdc.gov.tw/En/Bulletin/Detail/R1K7gSjoYa7Wojk54nW7fg?typeid=158.
4. Taiwan Center for Disease Control (CDC). CECC raises epidemic warning to Level 3 nationwide from May 19 to May 28; strengthened measures and restrictions introduced across Taiwan to reduce community transmission [Internet]. Taiwan CDC. 2021 [Cited 2021 September 07]. Available online: https://www.cdc.gov.tw/En/Bulletin/Detail/VN_6yeoBTKhRKoSy2d0hJQ?typeid=158.
5. Lee DK. Starting April 1, Face Mask Use Mandated for Intercity Transportation [Internet]. THE NEWS LENS. 2020 [Cited 2021 September 07].Available online: https://international.thenewslens.com/article/133261
6. Taiwan Center for Disease Control (CDC). CECC urges people subjected to home quarantine/isolation to follow related regulations to protect everyone’s health [Internet]. Taiwan CDC. 2021 [Cited 2021 September 07]. Available online: https://www.cdc.gov.tw/En/Bulletin/Detail/mhkl82LMg-mKGeltPu7JPw?typeid=158.
7. Taiwan Ministry of Health and Welfare. Accepted compensation applications for individuals under quarantine/isolation [Internet]. Taiwan Ministry of Health and Welfare. 2020[Cited 2021 October 1]. Available online: https://covid19.mohw.gov.tw/en/cp-4868-53898-206.html
8. Taiwan Center for Disease Control (CDC). Prevention and Control of COVID-19 in Taiwan [Internet]. Taiwan CDC. 2021 [Cited 2021 September 30]. Available online: https://www.cdc.gov.tw/En/Category/Page/0vq8rsAob_9HCi5GQ5jH1Q
9. Tourism Bureau, Taiwan. Taiwanstay, Accommodation. LEGAL ACCOMMODATION. [Cited 2021 September 30]. Available online: https://taiwanstay.net.tw/.
10. Tourism Bureau, Taiwan. Taiwanstay, Accommodation.Epidemic Prevention-Quarantine Hotels. [Cited 2021 October 1]Available online: https://taiwan.taiwanstay.net.tw/covhotel/.
11. Google LLC. COVID-19 Community Mobility Reports [Internet].GOOGLE. 2022 [Cited 2024 February 24] . Available online: https://www.google.com/covid19/mobility/
12. Jones SP. Imperial College London Big Data Analytical Unit and YouGov Plc. Imperial College London YouGov Covid Data Hub, v1.0, YouGov Plc. April 2020. [Cited 2022 September 20]. Available from: https://github.com/YouGov-Data/covid-19-tracker..
13. Taiwan Center for Disease Control (CDC). CECC raises epidemic alert level for Taipei City and New Taipei City to Level 3 and strengthens national restrictions and measures, effective from May 15 to May 28, in response to increasing level of community transmission [Internet]. Taiwan CDC. 2021 [Cited 2021 September 7]. Available online: https://www.cdc.gov.tw/En/Bulletin/Detail/R1K7gSjoYa7Wojk54nW7fg?typeid=158.
14. Our World in Data. Coronavirus (COVID-19) Vaccinations [Internet]. 2021 [Cited 2021 September 10]. Available online: https://ourworldindata.org/covid-vaccinations.
15. Liu Y. and Rocklöv J. The reproductive number of the Delta variant of SARS-CoV-2 is far higher compared to the ancestral SARS-CoV-2 virus. J Travel Med. 2021; 28:taab124. doi: 10.1093/jtm/taab124.
16. World Health Organization. Tracking SARS-CoV-2 variants [Internet]. 2021 [Cited 2021 September 07]. Available online: https://www.who.int/en/activities/tracking-SARS-CoV-2-variants/.
17. Davies NG, Abbott S, Barnard RC, Jarvis CI, Kucharski AJ, Munday JD, et al. Estimated transmissibility and impact of SARS-CoV-2 lineage B. 1.1. 7 in England. Science. 2021; 372:eabg3055. doi: 10.1126/science.abg3055.
18. Taiwan Center for Disease Control (CDC). 1922 Epidemic Prevention Expert [Internet]. 2021 [Cited 2021 October 04]. Available online: https://www.facebook.com/TWCDC/
