# Supplementary material for: Suppression of the alpha, delta, and omicron variants of SARS-Cov-2 in Taiwan
Source: PLoS One. 2024 Mar 18;19(3):e0300303. doi: 10.1371/journal.pone.0300303 (PMC10947699; doi:10.1371/journal.pone.0300303)
Supplement: Attachment — Submitted filename: Reviewers Attachments.docx [file pone.0300303.s002.docx]

PLOS ONE

PONE-D-23-29703 - [EMID:d1fc004c3e3e78de]

Title: Suppression of the alpha, delta, and omicron variants of SARS-Cov-2 in Taiwan.

**General Comments:**

This article shares Taiwan's experience in combating the Alpha and Delta virus strains, which have higher mortality rates despite the lack of a vaccine. In the face of the Omicron virus, which is highly contagious but has a relatively mild fatality rate, Taiwan has also gradually opened its borders and successfully conquered the Omicron epidemic. This article describes four key factors underlying Taiwan's success in controlling the spread of COVID-19: quick responses; effective control measures with new technologies and rolling knowledge updates; unity and cooperation among Taiwanese government agencies, private companies and organizations, and individual citizens; and Taiwanese self-discipline.

I think these experiences are worth sharing with other countries. The topic of this article falls within the scope of this journal. I have the following suggestions.

1. Taiwan’s reality is different from many other countries, in part because it only borders the sea. Nonetheless, the timeliness of border management measures (earlier introduction of entry restrictions and health screening) may have contributed to Taiwan's relatively low number of cases. This article does not explore this as the early introduction of border controls is mentioned but its impact is not discussed further or compared with other countries. I suggest the author compare the epidemic outcomes-related performance of Taiwan and other island countries. Please discuss whether island countries can help prevent the epidemic.
2. This article presented four factors for controlling the spread of COVID-19 in Taiwan. Nevertheless, the importance of mandatory interventions (contact tracing, quarantine, border control) versus non-mandatory interventions (use of masks, social distancing) was not discussed. Individual self-discipline may have played a smaller role than mandatory restrictions that can be backed-up by legal legislation. I suggest the author mention this in the discussion section. Some modelling studies may be added in the discussion, for example:

- The effect of preventing subclinical transmission on the containment of COVID-19: Mathematical modeling and experience in Taiwan. <https://www.sciencedirect.com/science/article/pii/S1551714420301798>
- Relative role of border restrictions, case finding and contact tracing in controlling SARS-CoV-2 in the presence of undetected transmission: a mathematical modelling study

<https://www.ncbi.nlm.nih.gov/pmc/articles/PMC10019421/>

3. A modelling analysis using Taiwan case data concluded that population-based interventions may have played a major role in Taiwan's initial elimination efforts and that case-based interventions alone were insufficient to control the epidemic. I suggest the authors add the findings in the discussion section.
   - Comparison of Estimated Effectiveness of Case-Based and Population-Based Interventions on COVID-19 Containment in Taiwan. <https://jamanetwork.com/journals/jamainternalmedicine/fullarticle/2778395>
4. Several articles have discussed Taiwan’s experience and potential lessons in combating COVID-19, and this article does not add to what has been published. It would be important to cite and compare previous works on the Taiwanese experience, i.e., in order to support some of the results presented in the manuscript. For example,

- Comparison of Estimated Effectiveness of Case-Based and Population-Based Interventions on COVID-19 Containment in Taiwan. <https://jamanetwork.com/journals/jamainternalmedicine/fullarticle/2778395>
- Fighting COVID‐19 through government initiatives and collaborative governance: the Taiwan experience. Public Administration Review, 80(4), 665-670.
- An American's perspective living through COVID-19 in Taiwan. Journal of the Formosan Medical Association, 119(12), 1884.

5. Contact tracing is considered an important strategy to curb the spread of COVID-19. While this is true, no relevant references are provided to support this confirmation. It would be interesting to discuss the transmissibility of COVID-19) to close contacts. For example, there is a report from Taiwan on the use of contact tracing and testing to assess COVID-19 transmission dynamics among the first 100 confirmed cases in the country.

- Contact tracing assessment of COVID-19 transmission dynamics in Taiwan and risk at different exposure periods before and after symptom onset. JAMA internal medicine, 180(9), 1156-1163.

6. The findings underscore the need for accurate, comprehensive contact tracing and testing. However, they also stressed that simply identifying and isolating symptomatic patients may not be enough to interrupt transmission, and that social distancing measures will also be needed. A modeling study also suggests that isolating all at-risk cases through contact tracing can control the outbreak.

- The effect of preventing subclinical transmission on the containment of COVID-19: Mathematical modeling and experience in Taiwan. <https://www.sciencedirect.com/science/article/pii/S1551714420301798>
