# Supplementary material for: Suppression of the alpha, delta, and omicron variants of SARS-Cov-2 in Taiwan
Source: PLoS One. 2024 Mar 18;19(3):e0300303. doi: 10.1371/journal.pone.0300303 (PMC10947699; doi:10.1371/journal.pone.0300303)
Supplement: Attachment — Submitted filename: Comment(PONE-D-23-29703).docx [file pone.0300303.s003.docx]

**PLOS ONE**

PONE-D-23-29703

Title: Suppression of the alpha, delta, and omicron variants of SARS-Cov-2 in Taiwan.

**General Comments:**

I read the manuscript “Suppression of the alpha, delta, and omicron variants of SARS-Cov-2 in Taiwan.” and I thank the authors and the journal for the possibility of reviewing this manuscript.

The authors describe Taiwan's responses to alpha, delta, and omicron variants. Taiwan has controlled its borders and implemented strict quarantine rules in the first two years of pandemic (2020 and 2021), keeping infection and deaths numbers low. Taiwan then gradually eased border measures in March 2023, when Taiwan faced a new variant of concern: Omicron. In this manuscript, four factors for controlling the spread of COVID-19 in Taiwan are presented. They are

(1) Quick response;

(2) Effective control measures and precise quarantine strategy;

(3) Individual self-discipline;

(4) Unity and cooperation.

The paper provides valuable perspective on Taiwan's success in controlling the spread of COVID-19, especially in the face of alpha and delta variants. I think this article can be improved by addressing the following issues. It would be beneficial for the authors to broaden their horizons and consider other infectious diseases that may cause pandemics in the future.

1. The abstract should include some methods and results, not just the background and conclusion.
2. The methods section lacks some important details that readers need to evaluate the methods used. For example, the authors analyzed the time to border closure and conducted one-sample test to compare between Taiwan and Non-Taiwan countries prior to vaccine introduction. It’s unclear how the time to border closure was calculated. Please provide more details.
3. More information is needed about the data sources and how the time-varying reproduction number (Rt) was calculated, including the model used to estimate Rt and any assumptions made in the analysis. The authors are suggested to provide link to the data sources and the code used for calculating Rt in order to encourage reproducibility of their results.
4. Taiwan adopted a COVID-zero approach in the first two years of pandemic (2020 and 2021), keeping infection and deaths low. I wonder how the COVID-zero approach affects the economy or social health in Taiwan.
5. In the discussion section, please indicate some of the limitations of your study.

Addressing these concerns would strengthen the paper's argument and provide a more comprehensive understanding of Taiwan's approach to controlling the spread of COVID-19.
